# (*E*)-1-Phenyl­ethanone semicarbazone

**DOI:** 10.1107/S1600536809025847

**Published:** 2009-07-11

**Authors:** Hoong-Kun Fun, Chin Sing Yeap, Mahesh Padaki, Shridhar Malladi, Arun M Isloor

**Affiliations:** a X-ray Crystallography Unit, School of Physics, Universiti Sains Malaysia, 11800 USM, Penang, Malaysia; b Department of Chemistry, National Institute of Technology-Karnataka, Surathkal, Mangalore 575 025, India

## Abstract

In the title compound, C_9_H_11_N_3_O, the benzene ring is disordered over two positions with refined occupancies of 0.922 (5) and 0.078 (5). The program *PLATON* [Spek (2009 ▶). *Acta Cryst.* D**65**, 148–155] recommends the solution in the space group *C*2/*m* with *a* = 7.3050 (3), *b* = 6.6745 (2), *c* = 18.3853 (6) Å and β = 96.986 (2)°. However, the large number of non-extinct reflections needed to be ignored if *C*2/*m* is chosen suggested that the space group is incorrect, even though the *R* values are lower than that for *P*2_1_/*c*. The semicarbazone group is essentially planar, with a maximum deviation of 0.046 (1) Å for one of the N atoms. The mean plane of the semicarbazone group forms dihedral angles of 33.61 (8) and 39.1 (9)° with the benzene ring of the major and minor components, respectively. In the crystal structure, mol­ecules are linked by inter­molecular N—H⋯O hydrogen bonds into extended chains along the *c* axis. The crystal structure is further stabilized by weak inter­molucular C—H⋯π inter­actions.

## Related literature

For hydrogen-bond motifs, see: Bernstein *et al.* (1995 ▶). For applications of semicarbazone derivatives, see: Chandra & Gupta (2005 ▶); Jain *et al.* (2002 ▶); Pilgram (1978 ▶); Warren *et al.* (1977 ▶); Yogeeswari *et al.* (2004 ▶). For the preparation of the title compound, see: Furniss *et al.* (1978 ▶). For related structures, see: Fun *et al.* (2009*a* ▶,*b* ▶,*c* ▶). For the stability of the temperature controller used for the data collection, see: Cosier & Glazer (1986 ▶).
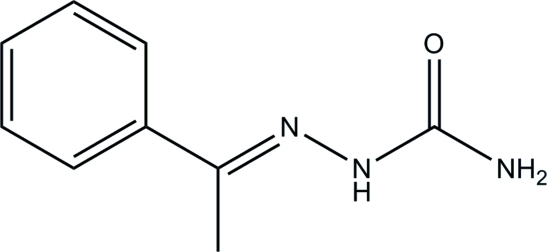


## Experimental

### 

#### Crystal data

C_9_H_11_N_3_O
*M* *_r_* = 177.21
Monoclinic, 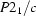

*a* = 18.3853 (6) Å
*b* = 6.6745 (2) Å
*c* = 7.3050 (3) Å
β = 96.986 (2)°
*V* = 889.76 (5) Å^3^
*Z* = 4
Mo *K*α radiation
μ = 0.09 mm^−1^
*T* = 100 K
0.32 × 0.13 × 0.03 mm

#### Data collection

Bruker SMART APEXII CCD area-detector diffractometer
Absorption correction: multi-scan (**SADABS**; Bruker, 2005 ▶) *T*_min_ = 0.881, *T*_max_ = 0.997
9294 measured reflections
2034 independent reflections
1449 reflections with *I* > 2σ(*I*)
*R*_int_ = 0.043

#### Refinement

*R*[*F* ^2^ > 2σ(*F* ^2^)] = 0.056
*wR*(*F* ^2^) = 0.181
*S* = 1.08
2034 reflections
148 parameters
H atoms treated by a mixture of independent and constrained refinement
Δρ_max_ = 0.43 e Å^−3^
Δρ_min_ = −0.46 e Å^−3^

### 

Data collection: *APEX2* (Bruker, 2005 ▶); cell refinement: *SAINT* (Bruker, 2005 ▶); data reduction: *SAINT*; program(s) used to solve structure: *SHELXTL* (Sheldrick, 2008 ▶); program(s) used to refine structure: *SHELXTL*; molecular graphics: *SHELXTL*; software used to prepare material for publication: *SHELXTL* and *PLATON* (Spek, 2009 ▶).

## Supplementary Material

Crystal structure: contains datablocks global, I. DOI: 10.1107/S1600536809025847/lh2857sup1.cif

Structure factors: contains datablocks I. DOI: 10.1107/S1600536809025847/lh2857Isup2.hkl

Additional supplementary materials:  crystallographic information; 3D view; checkCIF report

## Figures and Tables

**Table 1:** Hydrogen-bond geometry (Å, °)

*D*—H⋯*A*	*D*—H	H⋯*A*	*D*⋯*A*	*D*—H⋯*A*
N2—H1*N*2⋯O1^i^	0.88 (3)	2.02 (3)	2.901 (3)	177.2 (19)
N3—H2*N*3⋯O1^ii^	0.86 (3)	2.04 (3)	2.894 (3)	173 (3)
C2*A*—H2*AA*⋯*Cg*^iii^	0.93	2.93	3.707 (2)	142
C5*A*—H5*AA*⋯*Cg*^iv^	0.93	2.90	3.678 (2)	142

Symmetry codes: (i) 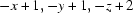; (ii) 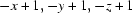; (iii) 
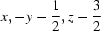
; (iv) 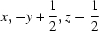. *Cg* is the centroid of the C1*A*,C2*A*,C3,C4*A*,C5*A*,C6 benzene ring.

**Fig. 1.**
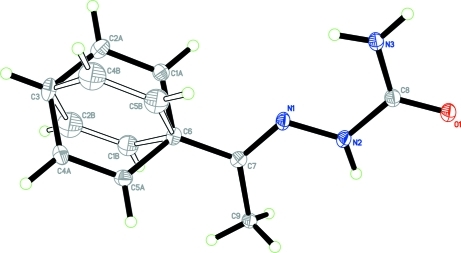
The molecular structure of the title compound with atom labels and 50% probability ellipsoids for non-H atoms. All disorder components are shown. The minor disorder component is shown with open bonds.

**Fig. 2.**
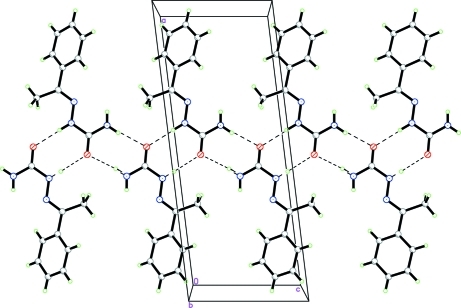
Part of the crystal structure of (I), viewed along the b axis. Intermolecular hydrogen bonds are shown in as dashed lines. Only the major disorder component is shown.

## supplementary crystallographic information

### Comment

In organic chemistry, a semicarbazone is a derivative of an aldehyde or ketone formed by a condensation between a ketone or aldehyde and semicarbazide. Semicarbazone find immense applications in the field of synthetic chemistry, such as medicinal chemistry (Warren *et al.*, 1977), organometalics (Chandra & Gupta, 2005), polymers (Jain *et al.*, 2002) and herbicides (Pilgram, 1978). 4-Sulphamoylphenyl semicarbazones were synthesized and were found to possess anticonvulsant activity (Yogeeswari *et al.*, 2004). We hereby report the crystal structure of a semicarbazone of potential commercial importance, (I).

The bond lengths and angles of the title compound (I), (Fig. 1) are comparable to related structures (Fun *et al.*, 2009a, b, c). A maximum deviation of -0.046 (1) Å for atom N2 from atoms O1, N1, N2, N3, C6, C7, C8 and C9 indicates that the semicarbazone group is essentially coplanar. This mean plane makes dihedral angle of 33.61 (8) and 39.1 (9)° with benzene ring  of the major and minor component (C1A-C2A-C3-C4A-C5A-C6 and C1B-C2B-C3-C4B-C5B-C6), respectively. The molecules are linked together into infinite one-dimensional chains by the intermolecular N2—H1N2···O1^i^ and N3—H2N3···O1^ii^ (see Table 1 for symmetry codes) hydrogen bonds along the *c* axis (Fig. 2) and these hydrogen bonds generate *R*_2_^2^(8) ring motifs (Bernstein *et al.*, 1995). The crystal structure is stabilized by the weak intermolucular C—H···π interactions (Table 1).

### Experimental

Semicarbazide hydrochloride (1.0 g, 8.9 mmol) and freshly recrystallized sodium acetate (0.9 g, 10.9 mmol) were dissolved in water (10 ml) following a literature procedure (Furniss *et al.*, 1978). The reaction mixture was stirred at room temperature for 10 minutes. To this, (1.0 g, 8.32 mmol) acetophenone was added and shaken well. A little alcohol was added to dissolve the turbidity. It was shaken for 10 more minutes and allowed to stand. The semicarbazone crystallizes on standing for 6 h. The separated crystals were filtered, washed with cold water and recrystallized from alcohol. Yield: 1.37 g (93%). M.p. 473-478 K.

### Refinement

All hydrogen atoms were located in a difference Fourier map and refined freely. The benzene ring is disordered over 2 position with refined occupancies of 0.922 (5) and 0.078 (5). The program PLATON recommends the solution in *C*2/*m* space group with a = 7.3050 (3), b = 6.6745 (2), c = 18.3853 (6) Å and β = 96.986 (2)°. However the large number of non-extinct (i.e. observed) reflections needed to be ignored for the *C*2/*m* case suggested that the space group is incorrect even though the R-values are lower than that for *P*2_1_/*c*.

### Crystal data

**Table:**

C_9_H_11_N_3_O	*F*(000) = 376
*M**_r_* = 177.21	*D*_x_ = 1.323 Mg m^−^^3^
Monoclinic, *P*2_1_/*c*	Mo *K*α radiation, λ = 0.71073 Å
Hall symbol: -P 2ybc	Cell parameters from 2113 reflections
*a* = 18.3853 (6) Å	θ = 3.4–29.6°
*b* = 6.6745 (2) Å	µ = 0.09 mm^−^^1^
*c* = 7.3050 (3) Å	*T* = 100 K
β = 96.986 (2)°	Plate, colourless
*V* = 889.76 (5) Å^3^	0.32 × 0.13 × 0.03 mm
*Z* = 4	

### Data collection

**Table:**

Bruker SMART APEXII CCD area-detector diffractometer	2034 independent reflections
Radiation source: fine-focus sealed tube	1449 reflections with *I* > 2σ(*I*)
graphite	*R*_int_ = 0.043
φ and ω scans	θ_max_ = 27.5°, θ_min_ = 1.1°
Absorption correction: multi-scan (*SADABS*; Bruker, 2005)	*h* = −23→23
*T*_min_ = 0.881, *T*_max_ = 0.997	*k* = −8→8
9294 measured reflections	*l* = −5→9

### Refinement

**Table:**

Refinement on *F*^2^	Primary atom site location: structure-invariant direct methods
Least-squares matrix: full	Secondary atom site location: difference Fourier map
*R*[*F*^2^ > 2σ(*F*^2^)] = 0.056	Hydrogen site location: inferred from neighbouring sites
*wR*(*F*^2^) = 0.181	H atoms treated by a mixture of independent and constrained refinement
*S* = 1.08	*w* = 1/[σ^2^(*F*_o_^2^) + (0.0993*P*)^2^ + 0.2822*P*] where *P* = (*F*_o_^2^ + 2*F*_c_^2^)/3
2034 reflections	(Δ/σ)_max_ < 0.001
148 parameters	Δρ_max_ = 0.43 e Å^−^^3^
0 restraints	Δρ_min_ = −0.46 e Å^−^^3^

### Special details

**Table:**

Experimental. The crystal was placed in the cold stream of an Oxford Cyrosystems Cobra open-flow nitrogen cryostat (Cosier & Glazer, 1986) operating at 100.0 (1)K.
Geometry. All esds (except the esd in the dihedral angle between two l.s. planes) are estimated using the full covariance matrix. The cell esds are taken into account individually in the estimation of esds in distances, angles and torsion angles; correlations between esds in cell parameters are only used when they are defined by crystal symmetry. An approximate (isotropic) treatment of cell esds is used for estimating esds involving l.s. planes.
Refinement. Refinement of F^2^ against ALL reflections. The weighted R-factor wR and goodness of fit S are based on F^2^, conventional R-factors R are based on F, with F set to zero for negative F^2^. The threshold expression of F^2^ > 2sigma(F^2^) is used only for calculating R-factors(gt) etc. and is not relevant to the choice of reflections for refinement. R-factors based on F^2^ are statistically about twice as large as those based on F, and R- factors based on ALL data will be even larger.

### Fractional atomic coordinates and isotropic or equivalent isotropic displacement parameters (Å^2^)

**Table:**

	*x*	*y*	*z*	*U*_iso_*/*U*_eq_	Occ. (<1)
O1	0.51345 (7)	0.4963 (2)	0.7657 (2)	0.0165 (4)	
N1	0.32789 (9)	0.4925 (2)	0.8239 (3)	0.0130 (4)	
N2	0.40259 (9)	0.4938 (2)	0.8715 (3)	0.0142 (4)	
N3	0.41052 (10)	0.4917 (3)	0.5585 (3)	0.0174 (5)	
C3	0.05611 (11)	0.5011 (3)	0.7581 (3)	0.0169 (5)	
H3A	0.0064	0.5007	0.7151	0.020*	
C6	0.20605 (10)	0.5016 (3)	0.8879 (3)	0.0114 (4)	
C7	0.28592 (11)	0.5045 (3)	0.9528 (3)	0.0119 (5)	
C8	0.44581 (11)	0.4942 (3)	0.7296 (3)	0.0135 (5)	
C9	0.31370 (11)	0.5196 (3)	1.1541 (3)	0.0149 (5)	
H9A	0.3444	0.6357	1.1750	0.022*	
H9B	0.3415	0.4018	1.1920	0.022*	
H9C	0.2730	0.5311	1.2242	0.022*	
C1A	0.17941 (11)	0.3969 (3)	0.7290 (3)	0.0144 (5)	0.922 (5)
H1AA	0.2120	0.3267	0.6649	0.017*	0.922 (5)
C2A	0.10550 (12)	0.3954 (3)	0.6644 (3)	0.0176 (5)	0.922 (5)
H2AA	0.0888	0.3238	0.5584	0.021*	0.922 (5)
C4A	0.08190 (11)	0.6070 (3)	0.9162 (3)	0.0164 (5)	0.922 (5)
H4AA	0.0492	0.6784	0.9789	0.020*	0.922 (5)
C5A	0.15600 (11)	0.6073 (3)	0.9816 (3)	0.0142 (5)	0.922 (5)
H5AA	0.1726	0.6781	1.0883	0.017*	0.922 (5)
C1B	0.1548 (15)	0.395 (4)	0.979 (4)	0.022 (7)*	0.078 (5)
H1BA	0.1725	0.3268	1.0861	0.026*	0.078 (5)
C2B	0.0822 (18)	0.385 (5)	0.923 (5)	0.032 (8)*	0.078 (5)
H2BA	0.0506	0.3096	0.9861	0.039*	0.078 (5)
C4B	0.1048 (17)	0.607 (5)	0.664 (5)	0.032 (8)*	0.078 (5)
H4BA	0.0876	0.6756	0.5570	0.038*	0.078 (5)
C5B	0.1785 (16)	0.612 (5)	0.728 (4)	0.025 (7)*	0.078 (5)
H5BA	0.2103	0.6871	0.6662	0.030*	0.078 (5)
H1N2	0.4271 (15)	0.500 (3)	0.983 (4)	0.025 (7)*	
H1N3	0.3627 (14)	0.495 (3)	0.543 (4)	0.019 (6)*	
H2N3	0.4366 (14)	0.496 (3)	0.469 (4)	0.020 (6)*	

### Atomic displacement parameters (Å^2^)

**Table:**

	*U*^11^	*U*^22^	*U*^33^	*U*^12^	*U*^13^	*U*^23^
O1	0.0110 (7)	0.0241 (8)	0.0143 (8)	−0.0017 (6)	0.0015 (6)	−0.0005 (6)
N1	0.0088 (8)	0.0142 (8)	0.0159 (9)	0.0007 (6)	0.0010 (6)	−0.0003 (7)
N2	0.0094 (8)	0.0207 (9)	0.0121 (9)	−0.0008 (7)	−0.0002 (7)	0.0002 (8)
N3	0.0102 (9)	0.0303 (11)	0.0118 (9)	−0.0010 (8)	0.0020 (7)	0.0001 (8)
C3	0.0101 (9)	0.0172 (10)	0.0227 (12)	0.0015 (8)	−0.0007 (8)	0.0033 (9)
C6	0.0110 (9)	0.0101 (9)	0.0131 (10)	0.0001 (7)	0.0008 (7)	0.0028 (8)
C7	0.0131 (9)	0.0088 (9)	0.0135 (10)	0.0005 (7)	0.0006 (8)	0.0005 (8)
C8	0.0121 (9)	0.0133 (9)	0.0152 (10)	0.0000 (7)	0.0024 (7)	−0.0005 (8)
C9	0.0118 (9)	0.0243 (11)	0.0086 (10)	−0.0009 (8)	0.0016 (7)	−0.0015 (8)
C1A	0.0135 (10)	0.0140 (11)	0.0155 (12)	0.0018 (8)	0.0015 (9)	−0.0016 (9)
C2A	0.0158 (11)	0.0169 (11)	0.0189 (12)	−0.0007 (9)	−0.0028 (9)	−0.0009 (9)
C4A	0.0127 (11)	0.0186 (12)	0.0185 (12)	0.0018 (9)	0.0045 (9)	−0.0008 (9)
C5A	0.0160 (11)	0.0143 (11)	0.0120 (11)	0.0012 (9)	0.0011 (8)	−0.0008 (9)

### Geometric parameters (Å, °)

**Table:**

O1—C8	1.240 (2)	C6—C7	1.487 (3)
N1—C7	1.290 (3)	C7—C9	1.500 (3)
N1—N2	1.375 (2)	C9—H9A	0.9600
N2—C8	1.380 (3)	C9—H9B	0.9600
N2—H1N2	0.88 (3)	C9—H9C	0.9600
N3—C8	1.336 (3)	C1A—C2A	1.383 (3)
N3—H1N3	0.87 (3)	C1A—H1AA	0.9300
N3—H2N3	0.85 (3)	C2A—H2AA	0.9300
C3—C4B	1.39 (3)	C4A—C5A	1.388 (3)
C3—C4A	1.387 (3)	C4A—H4AA	0.9300
C3—C2A	1.394 (3)	C5A—H5AA	0.9300
C3—C2B	1.46 (3)	C1B—C2B	1.35 (4)
C3—H3A	0.9300	C1B—H1BA	0.9300
C6—C1A	1.392 (3)	C2B—H2BA	0.9300
C6—C5A	1.402 (3)	C4B—C5B	1.38 (4)
C6—C1B	1.41 (3)	C4B—H4BA	0.9300
C6—C5B	1.42 (3)	C5B—H5BA	0.9300
			
C7—N1—N2	118.86 (18)	N3—C8—N2	116.34 (18)
N1—N2—C8	117.31 (18)	C7—C9—H9A	109.5
N1—N2—H1N2	128.1 (18)	C7—C9—H9B	109.5
C8—N2—H1N2	114.6 (18)	H9A—C9—H9B	109.5
C8—N3—H1N3	119.0 (17)	C7—C9—H9C	109.5
C8—N3—H2N3	117.3 (18)	H9A—C9—H9C	109.5
H1N3—N3—H2N3	124 (2)	H9B—C9—H9C	109.5
C4B—C3—C4A	88.6 (14)	C2A—C1A—C6	121.3 (2)
C4B—C3—C2A	61.1 (13)	C2A—C1A—H1AA	119.4
C4A—C3—C2A	119.32 (19)	C6—C1A—H1AA	119.4
C4B—C3—C2B	120.7 (19)	C1A—C2A—C3	120.0 (2)
C4A—C3—C2B	62.5 (13)	C1A—C2A—H2AA	120.0
C2A—C3—C2B	88.3 (13)	C3—C2A—H2AA	120.0
C4B—C3—H3A	120.2	C3—C4A—C5A	120.6 (2)
C4A—C3—H3A	120.3	C3—C4A—H4AA	119.7
C2A—C3—H3A	120.3	C5A—C4A—H4AA	119.7
C2B—C3—H3A	119.1	C4A—C5A—C6	120.4 (2)
C1A—C6—C5A	118.32 (18)	C4A—C5A—H5AA	119.8
C1A—C6—C1B	87.4 (12)	C6—C5A—H5AA	119.8
C5A—C6—C1B	60.4 (11)	C2B—C1B—C6	125 (3)
C1A—C6—C5B	61.3 (12)	C2B—C1B—H1BA	117.4
C5A—C6—C5B	87.2 (12)	C6—C1B—H1BA	117.4
C1B—C6—C5B	117.2 (17)	C1B—C2B—C3	116 (3)
C1A—C6—C7	120.39 (17)	C1B—C2B—H2BA	122.1
C5A—C6—C7	121.27 (19)	C3—C2B—H2BA	122.1
C1B—C6—C7	123.1 (12)	C5B—C4B—C3	121 (3)
C5B—C6—C7	119.7 (12)	C5B—C4B—H4BA	119.6
N1—C7—C6	114.93 (18)	C3—C4B—H4BA	119.6
N1—C7—C9	123.85 (18)	C4B—C5B—C6	120 (3)
C6—C7—C9	121.22 (17)	C4B—C5B—H5BA	119.9
O1—C8—N3	124.04 (19)	C6—C5B—H5BA	119.9
O1—C8—N2	119.63 (19)		
			
C7—N1—N2—C8	−175.79 (18)	C2B—C3—C4A—C5A	70.3 (14)
N2—N1—C7—C6	−179.91 (16)	C3—C4A—C5A—C6	0.5 (3)
N2—N1—C7—C9	−0.2 (3)	C1A—C6—C5A—C4A	−0.1 (3)
C1A—C6—C7—N1	30.7 (3)	C1B—C6—C5A—C4A	−68.7 (14)
C5A—C6—C7—N1	−147.66 (19)	C5B—C6—C5A—C4A	54.9 (12)
C1B—C6—C7—N1	139.4 (14)	C7—C6—C5A—C4A	178.31 (19)
C5B—C6—C7—N1	−41.4 (14)	C1A—C6—C1B—C2B	−54 (3)
C1A—C6—C7—C9	−149.0 (2)	C5A—C6—C1B—C2B	71 (3)
C5A—C6—C7—C9	32.6 (3)	C5B—C6—C1B—C2B	2(4)
C1B—C6—C7—C9	−40.4 (14)	C7—C6—C1B—C2B	−179 (2)
C5B—C6—C7—C9	138.9 (14)	C6—C1B—C2B—C3	−2(4)
N1—N2—C8—O1	179.65 (17)	C4B—C3—C2B—C1B	2(4)
N1—N2—C8—N3	−0.5 (3)	C4A—C3—C2B—C1B	−68 (2)
C5A—C6—C1A—C2A	−0.3 (3)	C2A—C3—C2B—C1B	57 (3)
C1B—C6—C1A—C2A	53.8 (12)	C4A—C3—C4B—C5B	54 (3)
C5B—C6—C1A—C2A	−69.3 (13)	C2A—C3—C4B—C5B	−71 (3)
C7—C6—C1A—C2A	−178.78 (19)	C2B—C3—C4B—C5B	−2(4)
C6—C1A—C2A—C3	0.4 (3)	C3—C4B—C5B—C6	2(4)
C4B—C3—C2A—C1A	69.9 (17)	C1A—C6—C5B—C4B	68 (3)
C4A—C3—C2A—C1A	−0.1 (3)	C5A—C6—C5B—C4B	−57 (3)
C2B—C3—C2A—C1A	−57.0 (13)	C1B—C6—C5B—C4B	−2(3)
C4B—C3—C4A—C5A	−55.7 (14)	C7—C6—C5B—C4B	179 (2)
C2A—C3—C4A—C5A	−0.4 (3)		

### Hydrogen-bond geometry (Å, °)

**Table:**

*D*—H···*A*	*D*—H	H···*A*	*D*···*A*	*D*—H···*A*
N2—H1N2···O1^i^	0.88 (3)	2.02 (3)	2.901 (3)	177.2 (19)
N3—H2N3···O1^ii^	0.86 (3)	2.04 (3)	2.894 (3)	173 (3)
C2A—H2AA···Cg^iii^	0.93	2.93	3.707 (2)	142
C5A—H5AA···Cg^iv^	0.93	2.90	3.678 (2)	142

Symmetry codes: (i) −*x*+1, −*y*+1, −*z*+2; (ii) −*x*+1, −*y*+1, −*z*+1; (iii) *x*, −*y*−1/2, *z*−3/2; (iv) *x*, −*y*+1/2, *z*−1/2.
